# Difluoromethylornithine (DFMO) and Neuroblastoma: A Review

**DOI:** 10.7759/cureus.37680

**Published:** 2023-04-17

**Authors:** Adarsh Vardhan Tangella, Ashwin S Gajre, Punith Chowdary Chirumamilla, Pemma V Rathhan

**Affiliations:** 1 Internal Medicine, Andhra Medical College and King George Hospital, Visakhapatnam, IND; 2 Internal Medicine, Lokmanya Tilak Municipal Medical College and Hospital, Mumbai, IND; 3 Internal Medicine, Guntur Medical College, Guntur, IND; 4 Internal Medicine, Osmania Medical College, Hyderabad, IND

**Keywords:** chemotherapy, neuro oncology, pediatric, dfmo, neuroblastoma

## Abstract

Neuroblastoma is a type of cancer that affects the sympathetic nervous system and is the most common extracranial solid tumor in children. Difluoromethylornithine (DFMO) is a drug that has shown promise as a treatment option for high-risk neuroblastoma. This review aims to provide an overview of the current research on the use of DFMO in neuroblastoma treatment. The review includes a discussion of the mechanisms of action of DFMO, as well as its potential for use in combination with other treatments such as chemotherapy and immunotherapy. The review also examines the current clinical trials involving DFMO in high-risk neuroblastoma patients and provides insights into the challenges and future directions for the use of DFMO in neuroblastoma treatment. Overall, the review highlights the potential of DFMO as a promising therapy for neuroblastoma and highlights the need for further research to fully understand its potential benefits and limitations.

## Introduction and background

Neuroblastoma arises in immature nerve cells called neuroblasts, which are found in the adrenal glands, neck, chest, and spinal cord. Neuroblastoma can be classified into different risk categories based on factors such as age at diagnosis, the extent of the disease, and genetic abnormalities. It is the most common solid tumor in children outside of the brain, with an incidence rate of approximately one in 7,000 live births [1-4]. Neuroblastoma is more common in children under the age of five years, and it rarely occurs in adults [1-4]. The incidence rate of neuroblastoma varies slightly depending on geographic location, ethnicity, and other factors. In the United States, it is estimated that about 800 new cases of neuroblastoma are diagnosed each year [1-4]. Overall, while neuroblastoma is a rare disease, it can be a serious and life-threatening condition, especially if it has spread to other parts of the body. Early diagnosis and treatment are important for improving the chances of a successful outcome [1-4].

Staging

The International Neuroblastoma Risk Group (INRG) staging system is a widely used system for staging neuroblastoma, a type of cancer that arises from immature nerve cells. The INRG staging system takes into account various factors, such as the location of the tumor, the extent of tumor spread, and certain genetic abnormalities, to categorize neuroblastoma into different stages. The stages of neuroblastoma according to the INRG staging system are summarized below [5]:

- Stage L1: This stage is used for infants younger than 12 months with localized tumors that can be completely resected (removed by surgery).

- Stage L2: This stage is used for infants younger than 12 months with tumors that are not completely resectable, but have not spread beyond the primary site.

- Stage M: This stage is used for patients older than 12 months with metastatic disease (spread to distant sites) or tumors that cannot be completely resected.

- Stage MS: This stage is used for patients of any age with metastatic disease that is limited to certain sites, such as the bone or bone marrow.

- Stage LMS: This stage is used for patients of any age with tumors that are completely resected but have genetic abnormalities that confer a high risk of relapse.

The INRG staging system provides a standardized way to classify neuroblastoma, which can help guide treatment decisions and predict outcomes [5].

High-risk neuroblastoma

High-risk neuroblastoma is a more aggressive and advanced form of neuroblastoma. It has a higher likelihood of spreading to other parts of the body and is associated with a poorer prognosis. The incidence of high-risk neuroblastoma is lower than that of neuroblastoma overall. According to the American Cancer Society, high-risk neuroblastoma accounts for about half of all neuroblastoma cases or approximately 400 new cases per year in the United States [1-4].

The criteria for high-risk neuroblastoma can vary slightly between different treatment protocols, but generally, it includes one or more of the following factors [4,5]:

- Age at diagnosis: Children older than 18 months at the time of diagnosis are considered to be at higher risk.

- Tumor stage: Advanced stages (stages 3 and 4) are associated with a higher risk of relapse and poorer outcomes.

- Tumor site: Tumors that originate in the abdomen, chest, or pelvis are associated with a higher risk of relapse and poorer outcomes.

- Tumor biology: Certain genetic abnormalities, such as MYCN amplification, are associated with a higher risk of relapse and poorer outcomes.

- Elevated levels of certain blood markers: Elevated levels of markers such as lactate dehydrogenase (LDH) and neuron-specific enolase (NSE) are associated with a higher risk of relapse and poorer outcomes.

It is important to note that not all children with high-risk neuroblastoma will have all of these risk factors, and some children with low-risk neuroblastoma may have one or more of these risk factors. Treatment decisions should be made on a case-by-case basis by a multidisciplinary team of healthcare professionals.

Established standard regimens available for the management of high-risk neuroblastoma

**Figure 1:**
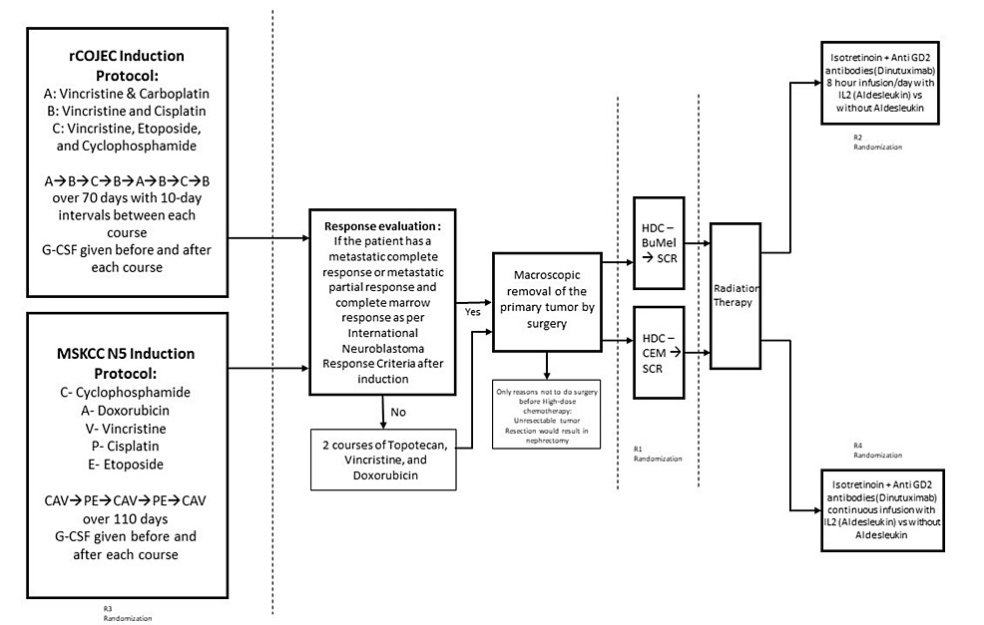
HR-NBL1.5 SIOPEN trial protocol (comparison of pre-existing HR-NBL1 SIOPEN vs. MSKCC N5 protocols)* *[9] R1: Busulfan melphalan (BuMel) vs. cyclophosphamide, etoposide, and melphalan (CEM) for high-dose therapy (HDT) followed by stem cell rescue (SCR) R2: Dinutuximab 8-hour infusion with aldesleukin vs. without aldesleukin (IL2) R3: rCOJEC vs. MSKCC N5 induction protocol R4: Dinutuximab continuous infusion with aldesleukin vs. without aldesleukin (IL2)

Physicians have always pondered the question of how to improve the overall survival (OS) and event-free survival (EFS) rates in high-risk neuroblastoma. There are three standard high-risk neuroblastoma regimens by three different organizations (COG - Children's Oncology Group [6], MSKCC - Memorial Sloan Kettering Cancer Centre [7,8], and SIOPEN - International Pediatric Oncology Society - Europe - Neuroblastoma [9,10]) that are being used widely now in the field of oncology. Each of them utilizes a wide range of treatment modalities that include chemotherapy, surgery, radiotherapy, high-dose chemotherapy with autologous stem cell transplant, targeted therapy, and immunotherapy. Targeted radionuclide therapy is also being investigated as an additional treatment approach along with existing modalities. High-Risk Neuroblastoma Regimen - SIOPEN 1.5 (HR-NBL 1.5/SIOPEN) was a study done where participants were randomized between the rCOJEC regimen (described in the flowchart Figure 1) and MSKCC N5 regimen at 4 different levels (Figure 1). The regimens have been summarized in Table 1.

**Table 1:** Standard regimens available for the management of high-risk neuroblastoma COG: Children's Oncology Group; MSKCC: Memorial Sloan Kettering Cancer Centre; SIOPEN: International Pediatric Oncology Society - Europe - Neuroblastoma

Study	Protocol	Result
ANBL0032 COG [6]	Induction - Response evaluation - Surgery - High-dose chemotherapy and stem cell rescue - Radiation therapy - Randomization between the isotretinoin-only arm and isotretinoin with dinutuximab (anti-GD2 antibodies) with aldesleukin (IL-2) and granulocyte-monocyte colony-stimulating factor (GM-CSF)	The use of dinutuximab + aldesleukin + GM-CSF + isotretinoin arm had improved event-free survival and overall survival over the isotretinoin arm
HR-NBL1/SIOPEN Induction Regimen (rapid COJEC) [10]	A-B-C-B-A-B-C-B over 70 days; A: vincristine, carboplatin; B: vincristine, cisplatin; C: vincristine, etoposide, and cyclophosphamide	
MSKCC N5 Induction Regimen [8]	CAV - PE - CAV - PE - CAV over 110 days; C: cyclophosphamide, A: doxorubicin, V: vincristine, P: cisplatin, E: etoposide	7 alternating cycles have no additional advantage over 5 alternating cycles
HR-NBL 1.5/SIOPEN [9]	(Flowchart below)	Comparison between HR-NBL1 (rCOJEC) and MSKCC N5 regimen – non-superiority of either over each other – yielded similar results

Cancer as a metabolic disease: rise in usage of metabolic enzyme inhibitors

As research has progressed, apart from the genetic basis for uncontrolled proliferation, scientists and physicians have identified that certain normal cellular metabolites have similar effects resulting in cellular hyperproliferation in cancers. The enzymes that control the synthesis of these metabolites have hence been implicated in cancers. Two such enzymes that have been commonly studied in relation to their effects on cancer cells are aldehyde dehydrogenase and ornithine decarboxylase (ODC). It was observed that the activity of these enzymes is elevated in certain cancers. Disulfiram, which is a potent aldehyde dehydrogenase inhibitor, was found to have anti-inflammatory and anti-cancer properties by multiple mechanisms [11]. A phase I clinical trial using a combination of liposomal doxorubicin and disulfiram is underway, which shall provide us with a better insight into this (Unique ID: NCT05210374).

DL-alpha-difluoromethylornithine, also known as DFMO, was discovered almost 40-45 years ago and is known for its ability to block ODC, an enzyme that produces polyamines (putrescine, spermidine, and spermine), which are then responsible for cell proliferation and the inhibition of apoptosis through a variety of cellular signaling pathways [12]. As a result, its application in diseases that show high rates of cytoproliferation became the main focus of research. In a lab, DFMO was applied to numerous cell lines, but the results were underwhelming [12]. It was not researched for a long time in malignancies due to its adverse effect profile, which includes reversible hearing loss and cytopenia [13].

Agents like DFMO were started to be used following the rise of the theory that cancer is a metabolic disease involving not only tumor suppressors or proto-oncogenes but also multiple single nucleotide polymorphisms (SNPs) in genes responsible for producing enzymes that are a part of physiological metabolic cascades. Recent research has revealed that tumor cells have increased activity of enzymes such as ODC and aldehyde dehydrogenase to promote proliferation and supply the required nutrients. This article provides a thorough analysis of the research on DFMO and how it can be used to treat neuroblastoma with or following the standard MSKCC/SIOPEN/COG regimen.

## Review

What is difluoromethylornithine (DFMO)?

DL-alpha-difluoromethylornithine, commonly known as DFMO or eflornithine, is a synthetic analog of the amino acid ornithine. It was created as an irreversible inhibitor of ODC, the first enzyme in the polyamine production pathway. Eflornithine was eventually shown to play a substantial role in the treatment of African sleeping sickness [14].

The parasite also showed an increased level of ODC when analyzed - which is probably the reason why DFMO works in cases of African sleeping sickness [14]. It has primarily been utilized as a backup for Trypanosoma brucei gambiense species, which are melarsoprol-resistant [14]. Eflornithine has been the gold standard of treatment for this condition for more than 50 years. Due to its trypanostatic activity rather than trypanocidal, the medication has a fairly slow onset of action [14].

Due to the higher levels and activity of ODC as well as the elevated amounts of polyamines, DFMO has been extensively researched on human neuroblastoma cell lines [15]. Moreover, alpha DFMO is alleged to cause total growth inhibition, cell body lengthening, and increased binding of antibodies against neuroectodermal antigens in human neuroblastoma cell lines [16].

Polyamines: synthesis, catabolism, and physiological effects

Polyamines have been known for a very long time since their discovery by microscopic observation of human semen. Chemically, they are polycations (positively charged molecules) that get attracted to and bind to negatively charged molecules such as DNA, RNA, or negatively charged proteins. Polyamines are of great interest due to their ability to impact cell proliferation, cellular aging, and death. The increase in polyamine concentrations in a lot of diseases ranging from cancer and psoriasis to parasitic infections has made it a very interesting target for disease management [17]. Polyamines have also been linked to Alzheimer’s disease [17].

The cascade for the synthesis of polyamines begins with the conversion of arginine to ornithine by arginase, which is a mitochondrial enzyme. Ornithine undergoes decarboxylation by ODC, which leads to the formation of putrescine - the first polyamine in the cascade. ODC expression is tightly regulated in the cell due to its cytoproliferative physiological effect. It is regulated at the levels of transcription and post-translation. ODC antizyme - which is a counter enzyme produced in cases of excess ODC expression - directly inhibits it and acts as an important checkpoint in the process of synthesis of polyamines [18].

**Figure 2:**
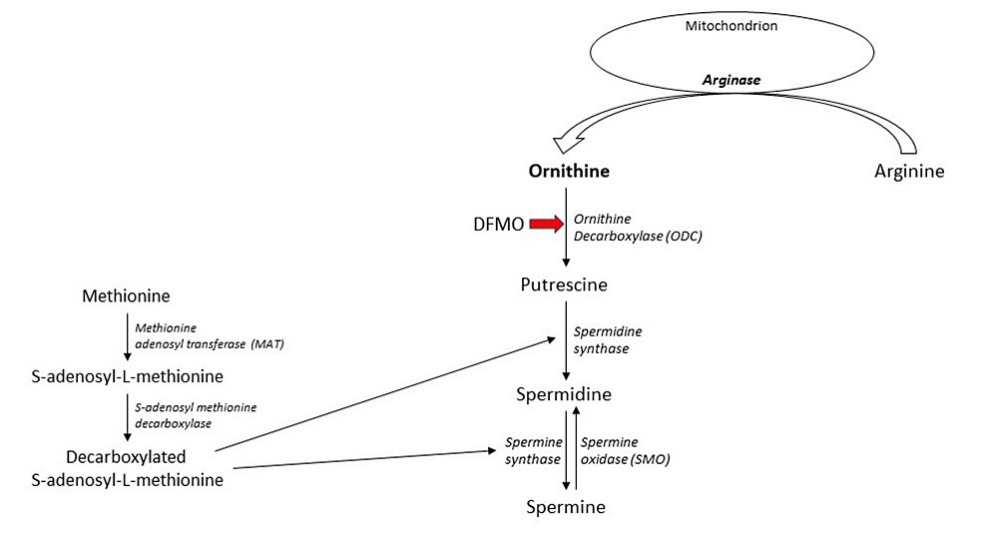
Synthesis of polyamines* *[18]

Parallel to this process, methionine is converted to S-Adenosyl methionine (SAM), which in turn is decarboxylated to decarboxylated S-Adenosyl methionine (De-SAM). This De-SAM is also an important product that influences polyamine synthesis as it is the aminopropyl group donor either to putrescine by spermidine synthase (SRM) or to spermidine by spermine synthase (SMS). Figure 2 illustrates the polyamine synthesis pathway [18].

**Figure 3:**
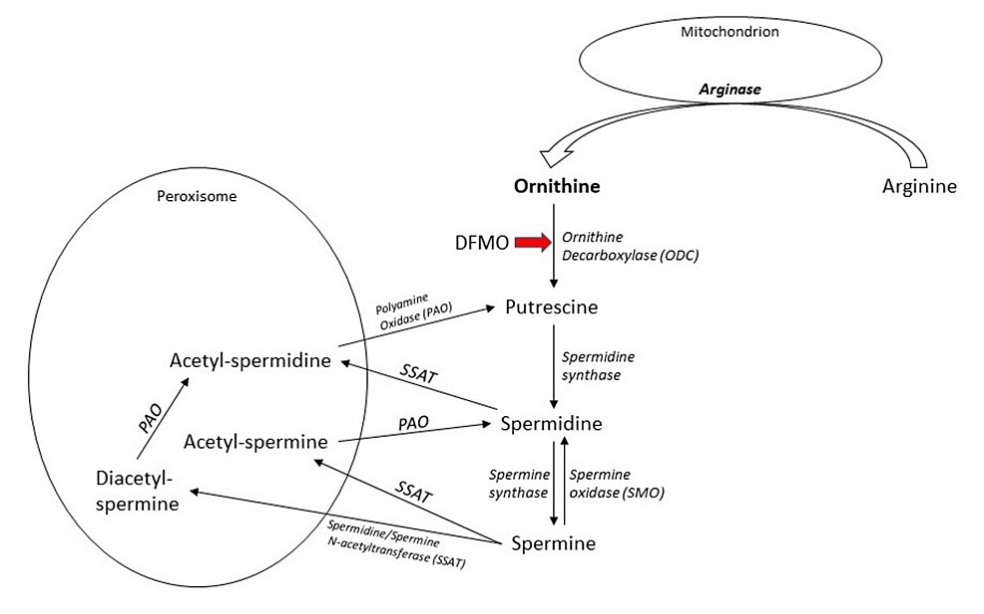
Catabolism of polyamines* *[18] PAO: polyamine oxidase; SSAT: spermidine/spermine acetyltransferase

The site of catabolism of polyamines is primarily in the peroxisomes. The major rate-limiting enzyme for this process is spermidine/spermine acetyltransferase (SSAT). It is involved in the conversion of spermine and spermidine into acetyl-spermine and acetyl-spermidine. The other major enzyme involved in the catabolism of polyamines is polyamine oxidase (PAO). PAO can convert the acetylated forms of spermidine and spermine to putrescine and spermidine respectively. Hence, higher polyamines get converted to putrescine by these enzymes. Spermine can be converted back to spermidine by spermine oxidase (SMO) in the cytoplasm. The notable byproducts of this process are hydrogen peroxide, which is the reason why peroxisomes need to be involved for biological clearance of this active chemical which, if not removed, can cause extensive free-radical injury to the cell [18]. Figure 3 depicts the polyamine catabolism pathway.

Polyamines play an important role in the process of aging. It has been observed that the levels of polyamines (especially putrescine and spermidine) decline with age. Along with the fall of polyamine levels, the activity of ODC also goes down drastically, thereby causing cellular aging due to reduced proliferation and, in turn, leading to decreased cell turnover [19]. But this goes against the fact that cancers in humans tend to have a higher incidence in older ages than younger age groups, proving that cancer biology and tumorigenesis are multifactorial and unregulated polyamine synthesis is just one of the driving factors. This is also probably why ODC irreversible inhibitors like DFMO tend to have less to no benefit if used as upfront single-agent chemotherapy but provide better results if used either in combination or as maintenance therapy following standard upfront chemotherapeutic regimens.

It is interesting to note that polyamines can have both anti-proliferative and proliferative actions on a cell in physiological states. They are involved in processes such as cellular aging, autophagy, and apoptosis. The main mechanism by which apoptosis gets triggered is by alteration of mitochondrial membrane potential. Polyamines cause an increase in calcium concentration inside mitochondria leading to pro-apoptotic signaling and activation of the BAX-BAK channel, which leads to the leakage of cytochrome c triggering the mitochondrial pathway of apoptosis [20]. It is also said that polyamines can directly cause the expulsion of cytochrome c into the cytoplasm, setting the cascade into motion [21]. Studies have noted that spermine specifically can cause a lowering of mitochondrial membrane potential, leading to cytochrome c release. This is a dose-dependent phenomenon. Spermine and putrescine can also cause uncoupling of the electron transport chain, leading to an ATP-depleted state in the cell, which results in cell death [22].

Due to the cationic nature of these molecules, polyamines can bind to nucleic acids which are anionic. Polyamine-DNA binding can lead to an alteration in the expression of genes. They can cause changes in the activity of histone acetylases and deacetylases [23]. The reduction of activity of histone acetylases and deacetylases was observed in the cells treated with polyamines in a lab [24]. Polyamines also have the ability to reduce necrosis mediated by cathepsin D.

The chief mechanism by which polyamines drive cells toward a hyperproliferative state is by activation of the MAPK pathway [25]. MAPK is linked to receptor tyrosine kinase on the cell membrane and influences the signal down streaming products of that pathway. BRAF and MEK are also a part of this pathway. Altogether, these genes tend to affect the expression of transcription factors like c-Myc which lead to cellular proliferation. C-Myc is a very important gene in the pathogenesis of neuroblastoma as it belongs to the family of protooncogenes and is associated with the gain of function mutations leading to hyperproliferation and eventually tumorigenesis.

Difluoromethylornithine (DFMO): animal-based laboratory studies and translational studies

ODC1 and MYCN Genes: The Relationship and How DFMO Can Break It

Although DFMO was discovered years ago, it was not studied for its use in cancers till the early 2000s. It had been completely restricted to its use in African sleeping sickness. Eventually, several laboratory studies showed a direct and statistically significant relationship between ornithine decarboxylase (ODC1) and MYCN gene expression, which led to the exploration of newer avenues for the use of DFMO - especially in neuroblastoma.

A study of particular interest here is by Hogarty et al., which was published in 2008 [26] and was a pre-clinical study. The main idea of the study is to identify the relationship between the MYCN gene and the myriad of ways it can cause the upregulation of polyamine synthesis. They also tried to study how ODC1 levels can be used as a prognostic factor in high-risk neuroblastomas. High-risk neuroblastomas constitute a majority of neuroblastoma cases diagnosed every year. High-risk neuroblastomas also usually express MYCN gain of function mutation, which is a proto-oncogene leading to uncontrolled cell proliferation. One of the most important conclusions from the study was that ODC1 expression correlates with survival in neuroblastoma. They have observed that ODC1 expression is significantly higher in MYCN-positive neuroblastomas and there was a strong significant correlation between MYCN expression and ODC1 expression (r=0.80, p<0.0001). Adverse EFS and OS were associated with tumors showing higher ODC1 expression. Since MYCN mutation can itself become a negative prognostic factor, Hogarty et al. have quantified the ODC1 expression in tumors without MYCN expression. The EFS and OS were worse in comparison to tumors with lower ODC1 expression, hence proving that ODC1 overexpression in itself can lower the prognosis. In addition to these, they have also found that the expression of ODC antizyme (OAZ2) is suppressed, leading to the unchecked activity of ODC. This was seen in both MYCN-amplified high-risk tumors (HR-A) and MYCN non-amplified high-risk tumors (HR-NA) [26].

Another notable experiment that is a part of this study was the upregulation of other enzymes that are a part of polyamine synthesis. SRM and adenosylmethionine decarboxylase (AMD1) showed increased levels of expression in MYCN-amplified tumors. SMS, which did not have any evidence that it is a target for Myc in the past, showed a strong correlation with MYCN amplification (r=0.42, p<0.0001). This study hence described one of the complex intracellular pathways by which MYCN can influence polyamine synthesis. When DFMO was added to neuroblastoma cell lines as a part of the same study, there was growth inhibition mediated by ODC1 suppression and was observed in cells independent of MYCN amplification. Cells demonstrated cytostasis (and not cytolysis) in the presence of DFMO [22].

While analyzing the effects of DFMO on MYCN amplified vs. non-amplified tumors in rats, it was observed that in tumors that arose from homozygous MYCN mutation, tumors were seen in the ganglia of mice; but when DFMO was used, the latency (31+/12 versus 43 ±7 days; p<0.001) and OS (mean 43 ±4 versus 59 ±9 days; p<0.001) were significantly higher than without DFMO. A very crucial conclusion here was that in hemizygous MYCN amplification, after DFMO therapy for 70 days, no tumors evolved. This proves that ODC1 inhibition by DFMO provides a long-lasting response. DFMO also inhibited progression and prolonged the time to death in mice [26].

When DFMO was coupled with other chemotherapeutic agents such as cisplatin, vincristine, or cyclophosphamide and when they were used to treat the tumors in mice, there was a marked increase in a relapse-free period, which was maximum with cyclophosphamide (almost increased OS to 80%). A major fear that arose out of these conclusions was the nature of neuroblastomas that occur even after using DFMO (if they have circumvented the polyamine synthesis inhibition). DFMO-treated and relapsed tumors in mice were examined and they showed poorly differentiated cellular architecture with areas of necrosis and hemorrhage. The ki67 index was also higher in these tumors. But, the rate of growth and the mitosis indices were similar to those seen in DFMO-responsive tumors [26].

The Let-7 miRNA/Lin28 Pathway, Ornithine Decarboxylase (ODC) Gene, and Influence of DFMO

**Figure 4:**
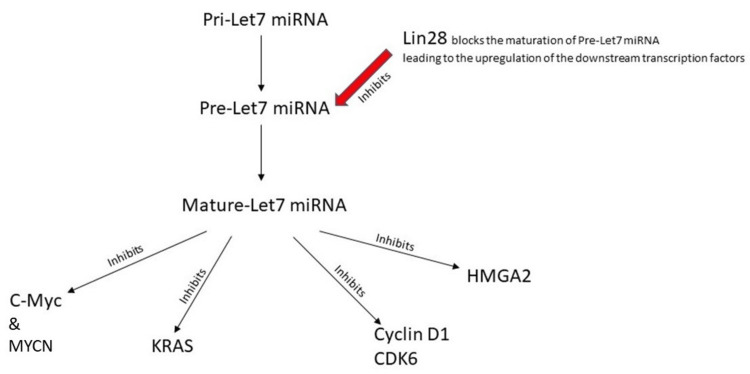
Let-7 miRNA/LIN28 pathway

Embryonic stem cells have a fairly simple mechanism for the regulation of proliferation, which helps in achieving a perfect balance between proliferation and differentiation. The Let-7 miRNA is produced in the nucleus as primary Let-7 miRNA and undergoes a two-step differentiation process to form pre-Let-7 miRNA and eventually mature to Let-7 miRNA. This Let-7 miRNA aids in post-transcriptional suppression of the products of these genes, ultimately helping in putting these oncogenes under check (Figure 4 shows the processing and effects of Let-7 miRNA). But there is also a need for proliferation. So, it was postulated that even Let-7 miRNA must have an inhibitor in place and the interaction between these two is like a see-saw balance causing controlled proliferation and differentiation. Upon further research, this protein which inhibits Let-7 miRNA was identified as Lin28 [27,28,29].

The next study reviewed here talks about how targeting ODC can help in the inhibition of glycolytic mechanisms in neuroblastoma. As shown in a figure later, ODC - through the Lin28 pathway - can create a Warburg-like phenomenon leading to increased glucose uptake by the cells which supplies the needed fuel for glycolysis, thereby helping the cell to produce more ATP. BE (2)-C, SMS-KCNR, and CHLA90 cell lines were treated with DFMO in-vitro. It was observed that the cell lines which expressed higher levels of Lin28B/MYCN were more sensitive to DFMO. CHLA90 cell line, which had the lowest Lin28B/MYCN fold change and needed a longer period of DFMO treatment to show a fall in Lin28B/MYCN expression [30].

The study also showed that these cell lines showed a reduced Lin28B and MYCN expression after being treated with DFMO, providing concrete evidence that ODC expression is directly related to the proliferative capacity of the cell. DFMO sensitive cell lines (BE (2)-C, SMS-KCNR) showed a more rapid fall in Lin28B/MYCN levels than CHLA90. A very important conclusion of this study was also that the DFMO-sensitive cell lines showed a rise in Let-7 miRNA levels after treatment with DFMO, which might also help in a sustained response as Let-7 miRNA is involved with the inhibition of various downstream transcriptional factors as described in a figure later [30].

**Figure 5:**
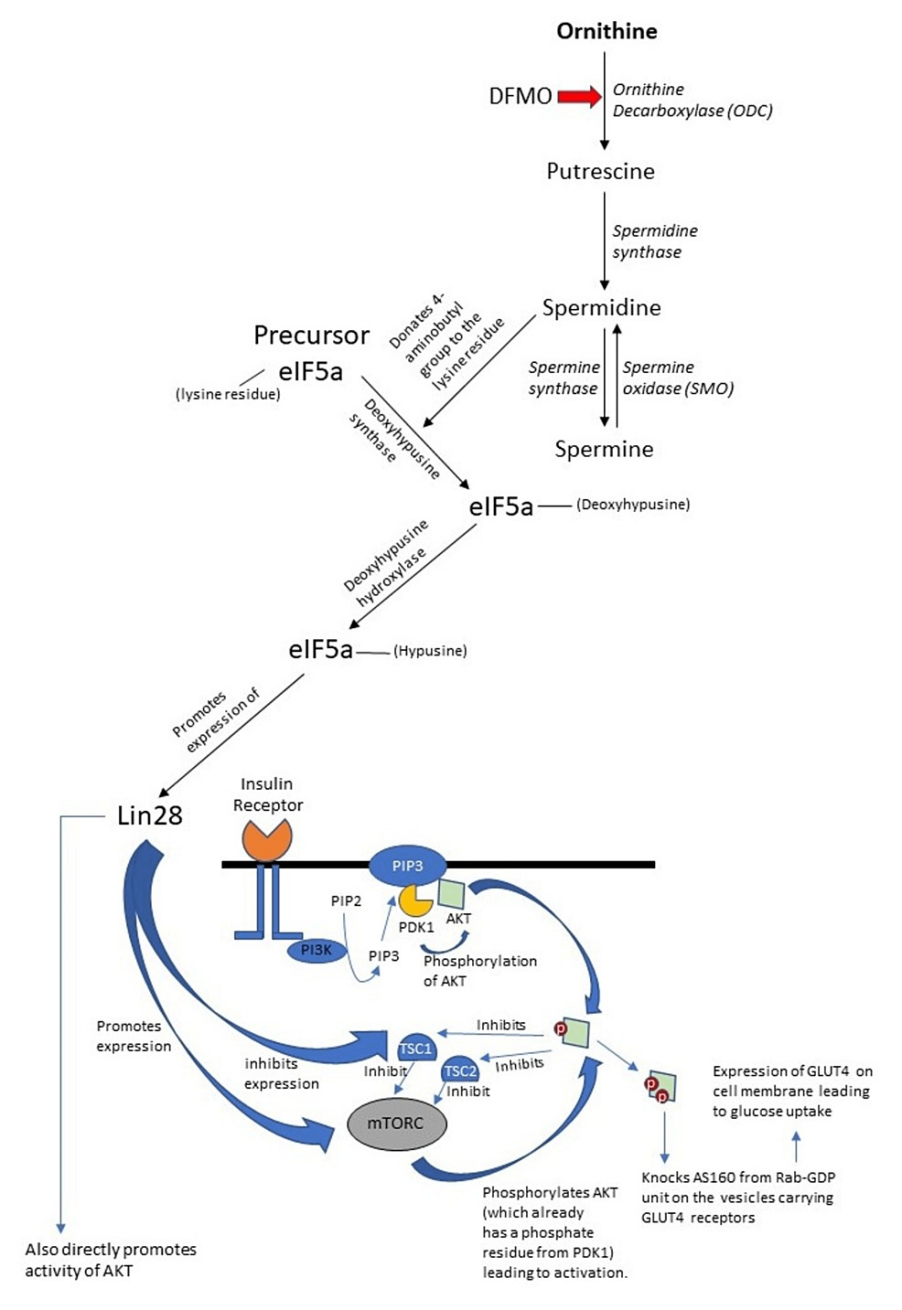
Flowchart showing Warburg-like effect by ODC-Lin28-AKT pathway

Since DFMO is causing a decrease in Lin28B expression, it indirectly also suppresses the activity of mTORC and AKT, leading to a suppression of glucose uptake via the insulin-PI3K-mTORC pathway (Figure 5 describes the pathways involved in this process). This was proved when the DFMO-sensitive cell lines showed lower ATP levels when compared to the DFMO-insensitive cell line. Since the glucose uptake was lowered, glycolytic metabolism also gets lowered and this was evident when PET-CT showed decreased uptake in the cells which - before DFMO therapy - were FDG avid [30].

Let-7 miRNA took the centre stage in a study done by Powers et al. where they tried to understand the mechanisms by which MYCN amplification influenced Let-7 miRNA levels in neuroblastoma cells [31]. Two interesting findings from this study are the possibility of ceRNA induced by MYCN which acts like a Let-7 miRNA sponge and the role of chromosomal deletions in neuroblastoma. Competing endogenous RNA is a group of RNA molecules that are induced by certain genes which bind to the miRNA associated with post-transcriptional repression of that specific gene and suppress its activity. This leads to partial or complete elimination of post-transcriptional firewalls for gene expression. In this case, they have identified various ceRNAs induced by MYCN and caused suppression of Let-7 miRNA leading to unregulated MYCN expression. It was also hypothesized that there is a possibility that MYCN mRNA itself can be a miRNA sponge without the need for induction of ceRNA [31]. Apart from MYCN amplification, chromosomal deletions have also been implicated in the pathogenesis of neuroblastoma. Chromosome 3p and 11q deletions have been found to be associated with neuroblastoma. Loss of chromosomes 3p and 11q resulted in the loss of Let-7 alleles causing a reduction in Let-7 miRNA levels in the cells [31,32,33].

E-box Sequences - MYCN - Ornithine Decarboxylase (ODC) Gene Single Nucleotide Polymorphisms and Difluoromethylornithine (DFMO)

Myc is a transcription-activating factor that has a profound effect on the proliferation of cells. It has been observed that Myc has a very specific sequence of amino acids to which it binds, causing the activation of enhancer boxes. CACGTG is the sequence to which Myc shows high affinity [34]. Myc also causes the transactivation of synthetic CACGTG sequences in mammalian motifs [35]. It is interesting to note that ODC genes are seen at various regions in mammalian DNA but only one of them codes for the enzyme [36]. A study was done to prove that the Myc family is a potent transactivator of ODC. To compare the binding of Myc to the CACGTG sequence, site-directed mutagenesis was done to alter the sequence to CACCTG from CACGTG. This showed a drastic lowering of Myc binding and reduced ODC transactivation [37]

The upstream region of the ODC gene has a promoter sequence, enhancer boxes with CACGTG sequence, and binding sites for transcription factors. The transcription activating region is a basic helix-loop-helix model with a leucine zipper (B-HLH-LZ). As a part of the study performed to evaluate the Myc transactivation of ODC, the role of the leucine zipper was also studied. Leucine residues were replaced with tryptophan and serine, which showed no altered levels of activation when compared to the wild type. But when leucine was replaced with proline, there was a significant reduction in the downstream ODC gene transcription [37].

Another important decisive factor for the effect of MYCN binding on ODC expression is ODC SNPs. The sequence between enhancer boxes that are responsible for ODC activation carries distinct single nucleotide polymorphisms, which have been found to have prognostic benefits in other cancers like colorectal and breast [38]. A study was done by Guo et.al to understand the effects of an SNP at +317 position in intron 1 upstream of the ODC gene. Upon performing a PCR-RFLP study, it was identified that an A/G polymorphism was present in the sequence flanked between the Myc binding regions (enhancer boxes). It was three bases upstream from the second enhancer box and hence it was further investigated to understand if there was any effect of this polymorphism on the ability of c-Myc to cause enhanced ODC transactivation. Luciferase reporter assays were done to understand this and proved that when the A allele was replaced with the G allele, there was a great reduction in luciferase induction. This proved that the strength of activation of ODC was indeed dependent on the SNP located at +317 close to the CACGTG c-Myc binding enhancer box [39].

**Figure 6:**
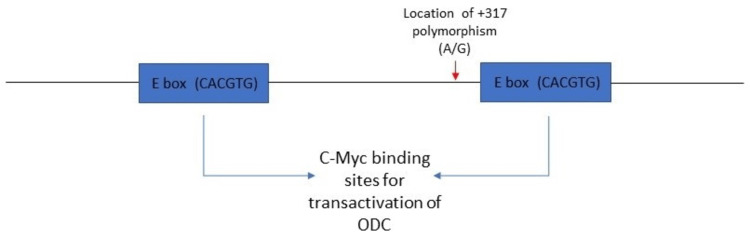
SNP located ODC gene and its influence on c-Myc activity* *[39] ODC: ornithine decarboxylase; SNP: single nucleotide polymorphism

The identification of an SNP that might be associated with enhanced ODC activation leads to the hypothesis that this allele might be associated with the alteration of EFS and OS indirectly as a consequence of the intensity of ODC activation. Phase 1 study done by Sholler et.al showed that the SNP affecting ODC expression, specifically the rs2302616 SNP, was associated with increased polyamines, enhanced susceptibility to the ODC inhibitor DFMO, and subsequently increased responsiveness to DFMO-containing therapies in patients with neuroblastoma (although the association of genotype with EFS did not reach statistical significance) [40]. But phase 2 study done by the same group showed that this SNP was not associated with any variation in EFS or OS [41]. Figure 6 is a line diagram showing the positions of the e-boxes and the SNP upstream from the ODC1 gene.

Hypothetical Reciprocity Between Myc Family and Ornithine Decarboxylase (ODC) Gene 

After trying to plot the downstream pathways regarding how ODC can influence Myc and how Myc has high-affinity sites (CACGTG) upstream from the ODC gene leading to enhanced ODC activation and transcription, it can be hypothesized that there is a possible cyclical reciprocal relationship between ODC and Myc.

ODC contributes to spermidine synthesis, which in turn plays a crucial role in eIF5a translation factor synthesis. eIF5a causes a Lin28-mediated activation of Myc (both c and N). This Myc can in turn reciprocally activate ODC. This piggyback activation, in the setting of a hyperproliferative state, can become a dangerous vicious cycle as both these genes can individually cause increased proliferation and together can lead to an amplified response.

**Figure 7:**
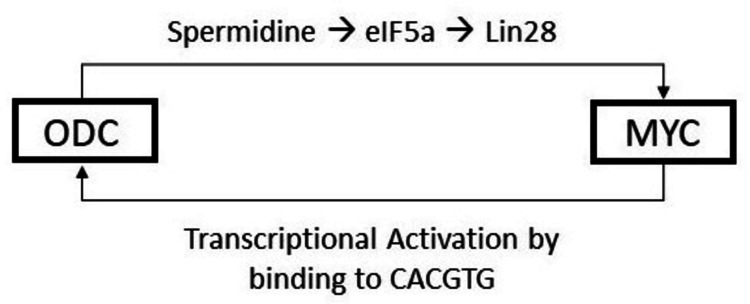
Reciprocity between ODC and MYC* *[26] ODC: ornithine decarboxylase

This hypothesis can be supported by the evidence regarding the response of MYCN levels in tumor cells that have been treated with DFMO. A study that has been discussed already has observed that ODC1 expression is significantly higher in MYCN-positive neuroblastomas and there was a strong significant correlation between MYCN expression and ODC1 expression (r=0.80, p<0.0001). A poorer EFS and OS were associated with tumors showing higher ODC1 expression [26]. Although this is a theoretical conclusion, this might play a crucial role in explaining the reason why adding DFMO as maintenance therapy in pediatric neuroblastoma could successfully increase EFS by almost 30% from an average of 55% to around 85%, thereby making ODC a very important target in the management of cancers with elevated polyamine metabolism and Myc mutations - which is seen in neuroblastoma. Figure 7 shows the reciprocity between ODC and MYC.

p27Kip1 and Difluoromethylornithine (DFMO)

Another important cell cycle regulator that was studied in detail regarding its response to DFMO in neuroblastoma is p27Kip1 (CDKN1A) [42].

**Figure 8:**
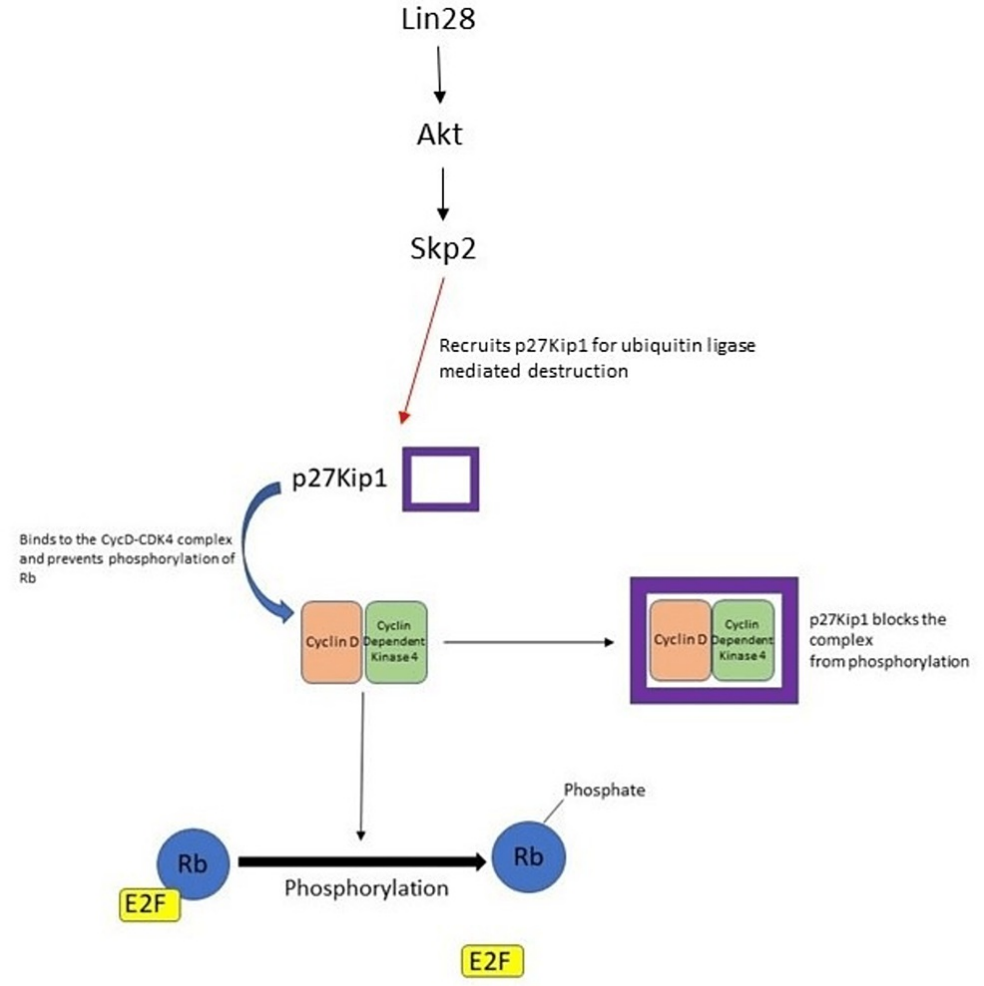
p27kip1 effect on cell cycle Rb: retinoblastoma gene

Lin28, which is linked to the polyamine pathway as mentioned earlier, causes Akt activation. Akt leads to increased expression of Skp2 (S phase kinase protein - which recruits substrates for ubiquitin ligases) [43]. Skp2 causes ubiquitin ligase-mediated destruction of phosphorylated p27Kip1, leading to unregulated cell proliferation (Figure 8 shows the effects of p27Kip1). As mentioned before, Lin28 expression is reduced by DFMO, and hence it was studied whether DFMO also has a role in increasing the intracellular levels of p27Kip1 leading to G1 phase cell cycle arrest.

Apart from its already well-known function of cell cycle regulation, recent studies on p27Kip1 have shown that it has a role in the regulation of cell migration and invasion, and metastasis. p27Kip1 stabilizes and binds to stathmin - which is a microtubule-stabilizing protein that is necessary for cellular motility and migration [44]. It also has a role in modulating RhoA activity, which is crucial for cellular invasion and migration.

In a study done by Koomoa et al., p27Kip1 role in neuroblastoma was studied in detail. There was a direct association between p27Kip1 levels of expression and survival - where patients whose tumors had high p27Kip1 had a survival rate of 70% up to 216 months and for those who had low p27Kip1 levels, the survival dropped to 0% by 30 months. The study also showed that there was a negative correlation between p27Kip1 and ODC expression (r= -0.216, p=0.04), which implies that higher levels of ODC drive the cell towards a hyperproliferative state by increasing polyamine levels and also by inhibiting important cell cycle regulators [42].

Another observation that was made was the higher levels of p27kip1 in non-metastatic cases of neuroblastoma when compared to those patients who have bone or bone marrow metastases. This proves that p27Kip1 levels have a direct relation to the ability of tumor cells to metastasize and when they are high, cell invasion and cell migration are lower, ultimately resulting in the prevention of diffuse metastases [42].

When they used neuroblastoma cell lines and subjected them to DFMO, the rate of proliferation dropped and it caused a G-1 phase arrest of the cells. Similar to the study described in the past, Koomoa et al. also identified that the inhibitory effect of DFMO correlates directly with the expression of MYCN. DFMO caused suppression of cell migration of both MYCN2-positive and MYCN2-negative cell lines by 73% and 72% respectively. This proves that the factors responsible for cellular metastases (p27kip1) operate independently from MYCN expression and DFMO can directly regulate the migration. To understand other processes such as NB invasion and invasive migration, the experiments were extended. NB migration was higher in cells with MYCN overexpression and DFMO inhibited this by 73% when compared to cells that were not treated with DFMO. DFMO also inhibited invasive migration in MYCN2-positive cells by 89% when compared to untreated cells. MYCN2+ cells, after being treated with DFMO, showed significantly reduced MYCN expression and also showed an increased accumulation of p27Kip1 levels. When the location of this accumulated p27Kip1 was studied, it was observed that p27kip1 accumulated both in the cytoplasm and nucleus, pointing to the dual functionality of it - cell cycle regulation and inhibition of cell invasion and migration wherein the nuclear fraction would help in cell cycle regulation and the cytoplasmic fraction would deal with inhibition of microtubule formation [42].

Clinical Trials: Difluoromethylornithine (DFMO) in Neuroblastoma

Sholler et al. have contributed a lot toward the rationale to include DFMO as maintenance therapy after completing standard regimens. They have successfully completed a phase I and a single arm prospective open phase II trial, which helped us understand the possible outcomes that can be expected if a patient with neuroblastoma is treated with DFMO. All the human studies done to evaluate the role of DFMO in neuroblastoma are summarized in a table later.

The phase I trial clearly showed that the toxicity profile of DFMO when used at a maximum dose of 1500 mg/m^2^ did not have any serious dose-limiting toxicities [40]. It can be taken in tablet form and the side effect profile is listed in a table later. 

The phase II trial was done mainly to find out if maintenance therapy with DFMO after successfully finishing a standard, internationally recognized regimen for neuroblastoma would have any role in improving the EFS and OS of patients (primary and secondary endpoints of the study respectively) with high-risk neuroblastoma (metastatic/high proliferative index/MYCN+ve/relapsed/refractory disease). They analyzed the two groups - one which successfully received a standard regimen and do not have any evidence of disease on MIBG/FGD PET and the other group that was treated with one or more runs of standard chemotherapeutic regimens and is refractory or has relapsed [41]. Table 2 compares EFS and OS in terms of previous data without DFMO to phase II trial with maintenance DFMO.

**Table 2:** Comparison of EFS and OS related to standard regimens vs. when maintenance DFMO was used EFS: event-free survival; OS: overall survival

Endpoint	Previous data-based	With maintenance DFMO [37]
		Stratum 1 Stratum 2
2-year event-free survival	66% ±5%	84% ±4% 54% ±8%
Overall survival	55-75%	97% ±2% 84% ±6%

A crucial point to be noted in this study is that 81% of the first group have also participated in the ANBL0032 trial, which involves the usage of dinutuximab, which is a chimeric anti-GD2 monoclonal antibody along with interleukin-2 (IL-2) receptor and isotretinoin, which did show improvement in EFS and OS [6]. Since ANBL0032 was a randomized trial, we do not know whether these subjects have received this regimen or not. This might point towards a possibility where the increased EFS in 81% of subjects of the first group might not be totally attributable to the anti-cancer effects of DFMO.

A question that would arise from this study would be about the role of DFMO along with other chemotherapeutic/immunotherapeutic/high-dose chemotherapy supported by autologous stem cell transplant. DFMO as a maintenance drug is the only aspect that was studied here, and hence another study is probably needed to look into the use of DFMO as a part of induction regimens in conjugation with other modalities.

An important investigation that could be done apart from the ODC SNP analysis is trying to analyze the Lin28/Let-7 miRNA pathway response and p27Kip1 levels in the tumor post-DFMO regimen to understand whether the changes that were seen in-vitro are also evident in the tumor specimens of these subjects. This would help us understand the possible role of directly targeting these pathways or the use of mTOR inhibitors (which are already available) along with DFMO as this would establish a multilevel blockade of the pathway that polyamines use to promote cellular proliferation. mTOR inhibitors (temsirolimus) have been studied in the setting of neuroblastoma but have not shown any significant treatment response except for meaningful prolonged stable disease. Hence, coupling them with DFMO would be a possible interest in the future [45]. Novel targets in neuroblastoma with references to their studies [45-55] have been summarized in Table 3 [56].

**Table 3:** Novel targets and drugs under investigation for neuroblastoma

Target	Drug
MEK	Trametinib, biminetinib [48]
AKT	Perifosine [49]
mTOR	Temsirolimus [45]
BCl2	Navitoclax
EGFR	Entrecitinib [50], afitinib [51], sorafenib [52]
B7-H3	Enoblituzumab [53]
GD2	Dinutuximab [54]
Somatostatin receptor	DOTATATE [55]
Norepinephrine transporter	I-131-MIBG [47]
CDK4/6 inhibitor	Palbociclib [46]

DFMO is a cytostatic and not a cytotoxic drug. It suppresses cellular proliferation. Hence the combination of DFMO with multiple chemotherapeutic regimens that are cytotoxic might also lead to better results and this is yet to be studied. Especially, the role of other drugs that actively suppress cytoproliferation such as CDK4/6 inhibitors in conjunction with DFMO should be studied. CDK4/6 inhibitors such as palbociclib did show a G1 phase arrest when neuroblastoma cell lines were treated with it in vitro hinting at a possible response in humans [46]

MIBG is another important chemical used in neuroblastoma to detect residual disease. It is a norepinephrine analog that helps detect the sites of active tumor masses in the body. But it can also be therapeutic due to its structural similarity with norepinephrine, especially when coupled with radioactive isotopes such as iodine-131. Theranostics in pediatric neuroblastomas is a growing interest. Although this regimen is known to have some notorious side effects, DFMO in addition to MIBG-based targeted radionuclide therapy [47] needs to be studied.

Another clinical trial was done to understand the efficacy of DFMO in combination with immunotherapy and targeted therapy. Targeted therapy was chosen depending on RNA sequencing of tumor/metastatic site samples acquired from the patients. The majority of them showed HDAC2 mutations and hence vorinostat was used the most (80%) followed by crizotinib (10%) as ALK was the second most mutated gene in this study pool of 20 people. The regimen included three to eight cycles of induction therapy following the COG ANBL0032 trial regimen wherein the targeted therapy was initiated in cycle three in the majority of cases. Peripheral blood stem cells (PBSC) were harvested during the induction phase and an autologous stem cell transplant was performed followed by radiation therapy. This was followed by the initiation of maintenance therapy with anti-GD2 antibodies (dinutuximab) with IL-2 and isotretinoin in alternating cycles. DFMO was added to the maintenance therapy and was continued up to two years post-cessation of immunotherapy. Genetic analysis showed overexpression of ODC1 in half of the patients and Lin28b overexpression in most of the patients which meant that DFMO can be beneficial in these patients. After the regimens for induction and immunotherapy were complete, five patients were identified to be in complete remission, five patients in very good partial response, and three showed partial response. The reason for discussing this study even though the two-year follow-up point after DFMO maintenance is not yet reached is that DFMO was also used as a part of the immunotherapy cycles. The study was published in 2022, and hence the results for DFMO two-year maintenance regimen probably will be out by 2024 [57].

An important aspect of this study is the genetic analysis of all the samples. As discussed earlier, the impact of DFMO is higher if there is MYCN mediated/non-mediated overexpression of ODC1 and/or if there is Lin28b overexpression as the crux of DFMO activity is by suppressing these pathways. This analysis of ODC1 and Lin28b along with p27kip1 levels might be beneficial if done before starting DFMO as the response can be quantified by the post-therapy expression of them or their products and this can also become a regular practice to give DFMO if the tumor is associated with upregulation of any of the above-mentioned genes which might be more beneficial.

As a part of understanding the impact of maintenance DFMO post-chemotherapy in high-risk neuroblastoma cases, Lewis et al. went on to compare the stratum I subset of the phase II trial (NMTRC003/003b) involving the use of maintenance DFMO (as discussed earlier) (n=81) to a control group (BCC001) that was made up of retrospective comparable data from previous trials that did not use DFMO as maintenance therapy (n=76) [58]. The difference in OS of patients with MYCN-amplified neuroblastoma showed a significant difference (p=0.027), which shows that MYCN-amplified tumors show a better response to DFMO over MYCN non-amplified tumors [54]. Table 4 shows a comparison of EFS and OS between NMTR0032/003b and the control group (BCC001). Table 5 presents a summary of studies done to evaluate the efficacy of DFMO in high-risk neuroblastoma.

**Table 4:** Comparison of EFS and OS between NMTRC003/003b (received maintenance DFMO) and BCC001 (retrospective data from BCC without DFMO maintenance)* *[58] EFS: event-free survival; OS: overall survival

Variable	NMTRC003/003b (n=81)	BCC001 group (n=76)
2-year EFS	86.4%	78.3%
5-year EFS	85.2%	65.6%
2-year OS	98.8%	94.4%
5-year OS	95.1%	81.6%

**Table 5:** Summary of studies done to evaluate the efficacy of DFMO in high-risk neuroblastoma EFS: event-free survival; PFS: progression-free survival; OS: overall survival

Study	Group	Year	EFS	PFS	OS
A phase I trial of DFMO targeting polyamine addiction in patients with relapsed/refractory neuroblastoma (NCT01059071) [36]	Sholler et al.	2015		Median PFS: 80.5 days	
Maintenance DFMO increases survival in high-risk neuroblastoma (phase II) (NCT01586260, NCT02395666) [37]	Sholler et al.	2018	Stratum 1: 2-year EFS 84%; stratum 2: 2-year EFS 54%		Stratum 1: 2-year OS 97%; stratum 2: 2-year OS: 84%
A subset analysis of a phase II trial evaluating the use of DFMO as maintenance therapy for high-risk neuroblastoma (stratum 1 from phase II vs. no maintenance DFMO) (NMTRC003/003b) [53]	Lewis et al.	2020	5-year EFS: 85.2%		5-year OS: 95.1%
A pilot study of genomic-guided induction therapy followed by immunotherapy with difluoromethylornithine maintenance for high-risk neuroblastoma (NCT02559778) [54]	Kraveka et al.	2022	Yet to reach 2-year or 5-year endpoint		Yet to reach 2-year or 5-year endpoint

Difluoromethylornithine (DFMO): Side Effect Profile

The side effect profile of DFMO was studied in detail as a part of the phase I trial done to understand its efficacy. We have tried to compare the risk of hematological side effects between periods when the participants received only DFMO versus DFMO with etoposide. Neutrophil count decrease showed a significant decrease with an OR of 6.65 (95% CI: 1.15-38.19, p=0.03). None of the other side effects showed a significant difference between the times when subjects received only DFMO vs. DFMO with etoposide. Table 6 compares the two regimens and lists all the side effects observed.

**Table 6:** Comparison of side effects between only DFMO cycle vs. DFMO with etoposide in NCT01059071* *[40] ALT: alanine transaminase; AST: aspartate transaminase; GGT: gamma glutamyl transferase; NR: not related

Side Effect	DFMO only (n=21)	DFMO + etoposide (n=17)	OR	95% CI	P-value
Anemia	3	5	2.5	0.52-12.46	0.26
Neutrophil count decrease	2	7	6.65	1.15-38.19	0.03^*^
Platelet count decrease	2	3	2.03	0.29-13.85	0.46
White blood cell decrease	0	1	NR	NR	NR
ALT elevation	1	1	NR	NR	NR
Anorexia	1	0	NR	NR	NR
AST elevation	1	2	NR	NR	NR
Conjunctivitis	1	0	NR	NR	NR
Constipation	1	1	NR	NR	NR
Diarrhea	1	0	NR	NR	NR
GGT elevation	1	0	NR	NR	NR
Hypoalbuminemia	1	0	NR	NR	NR
Hypophosphatemia	1	0	NR	NR	NR
Infection, sinus	0	1	NR	NR	NR
Mouth pain	0	1	NR	NR	NR
Nausea	0	1	NR	NR	NR
Neuropathy	0	1	NR	NR	NR
Pain	0	1	NR	NR	NR

As phase 1 is only for a limited period of time, side effects such as hearing loss were not observed, possibly pointing towards the idea that it is commonly caused by continuous prolonged intake of DFMO. Anemia and a decrease in platelet and neutrophil counts were the most common side effects observed. These side effects were more pronounced when the participants were taking DFMO along with etoposide, thus hinting that they can be due to etoposide or due to continuous intake of DFMO as the subjects were on multiple cycles of DFMO with etoposide but only a single cycle of DFMO alone.

Phase II study showed similar findings [41] where neutrophil count decrease, anemia, and ALT elevation were the most common side effects observed. Hearing loss was seen in 6/140 patients (about 5%), which supports the idea that hearing loss is more common when DFMO is taken for a long period of time. Side effects in the study where targeted therapy and immunotherapy were used were significantly higher when compared to using DFMO only, which could be due to the toxicity of targeted or immunotherapy agents by themselves potentiating the side effect profile of DFMO. The most common side effects observed were anemia and a platelet count decrease. Serious side effects such as febrile neutropenia were also seen in this cohort [57]. Table 7 compares the side effect profiles of the phase II study using maintenance DFMO vs. when targeted therapy and immunotherapy were added along with DFMO.

**Table 7:** Comparison of side effects of trials NCT01586260, NCT02395666* with those of NCT02559778** *[41], **[57] ALT: alanine transaminase; AST: aspartate transaminase; INR: international normalized ratio

Side effect	(NCT01586260, NCT02395666) (n=140) [41]	(NCT02559778) (n=20) [57]
Anemia	6	10
Neutrophil count decrease	11	10
Platelet count decrease	2	14
Febrile neutropenia	-	11
White blood cell decrease	3	7
Abdominal pain	1	-
Agitation	1	-
Alopecia	2	-
ALT elevation	12	1
AST elevation	9	1
Alkaline phosphatase elevation	1	-
Anorexia	1	1
Cellulitis	-	1
Dehydration	-	1
Diarrhea	7	1
Epistaxis	-	2
Fever	4	-
Hearing loss	6	-
Hypoglycemia	1	-
Hypokalemia	2	6
Hyponatremia	-	3
Hypophosphatemia	-	5
Infection (other)	3	4
Infection, middle ear	6	-
INR elevated	1	-
Insomnia	1	-
Nausea	-	4
Pain	2	-
Post-nasal drip	1	-
Rash	3	-
Sepsis	-	3
Skin infection	-	1
Thromboembolic event	-	1
Vomiting	1	3
Weight gain	1	-
Weight loss	-	1

Oncologists have been trying to find ways to extend EFS and enhance the quality of life for patients with high-risk neuroblastoma for years. The maximum EFS was only about 60% after using every available option, including immunotherapy, tandem autologous stem cell transplant, and chemotherapy, which is not much given that the time it takes for this disease to relapse is often shorter. A long-used medication that has been well studied for both its toxicity and effectiveness in people provided some light in the darkness on the possibility of boosting EFS by roughly 10-20%. Such a huge rise in EFS is extremely unusual, particularly in high-risk situations. But DFMO showed extraordinary results following its use in high-risk neuroblastoma. The benefits and convenience of use are its other advantages. It is an oral medication with fewer side effects than a lot of other drugs used to manage high-risk neuroblastoma, thereby improving patient compliance.

As of now, we are still awaiting the findings of the first combination chemotherapy experiment using DFMO. The patients’ and their family's overall quality of life is also diminished by high-risk neuroblastoma. There is a staggering level of physical and mental handicap that can be brought on by this disease and its psychosocial component. The very fact that this drug can give these kids a chance to have an almost normal childhood even if it is for a limited period of time matters a lot to their parents in the long run.

## Conclusions

DFMO represents a promising therapeutic option for high-risk neuroblastoma, a deadly childhood cancer that currently lacks effective treatments. Preclinical and clinical studies have demonstrated that DFMO can effectively inhibit ODC, the key enzyme in polyamine biosynthesis, leading to decreased tumor growth and improved survival in animal models and human patients. Moreover, DFMO appears to synergize with other chemotherapeutic agents, such as topotecan and irinotecan, to enhance their anti-tumor effects and reduce their toxicities. Despite these promising results, several challenges and questions remain regarding the optimal use of DFMO in high-risk neuroblastoma. For instance, the optimal dosing in various situations, duration, and combination with other therapies are not yet fully established, and the potential side effects of long-term DFMO use, such as hearing loss and renal toxicity, require careful monitoring and management. In addition, the mechanisms underlying the heterogeneity of DFMO response in different patients and tumors, as well as the potential resistance mechanisms and biomarkers of response, warrant further investigation.

Nonetheless, DFMO represents a prime example of the growing field of precision medicine, which aims to tailor treatments to the specific genetic and molecular characteristics of individual patients and tumors. By targeting the dysregulated polyamine pathway in high-risk neuroblastoma, DFMO offers a new avenue for personalized therapy and improved outcomes in this devastating disease. Future research should continue to explore the potential of DFMO in other cancers and in combination with emerging immunotherapies and targeted agents, as well as to refine its use in high-risk neuroblastoma.
